# Some Practical Notes

**Published:** 1909-09-18

**Authors:** 


### SOME PRACTICAL NOTES.

An extra outer cover should always be included in a motorist's equipment of spare parts, but it should be thoroughly protected against light, heat, oil, and dampness. One or two inner tubes should also be carried, and care should be taken to see that no deterioration takes place before they are put into use. To put an inner tube, uncovered, into a box full of loose tools, oil cans, etc., is only a little better than throwing it away. The tools will chafe and the oil will rot it, so that, if it holds air at all when inflated, it may soon burst under the weight of the car.

Should a car be in regular service it is better to leave the tyres inflated, but if the vehicle is not used for some months it is better, after having placed jacks under the axles, to deflate partially the tyres. If this is done it will add greatly to their life, as they are then bearing only the pressure of the air with which they are inflated, which is very slight, whereas when supporting the weight of the car this is exerting a continual unnecessary strain on the walls of the cover. By adopting this course it is estimated that the life of the tyres will be increased at least half the time the car stands idle.

If necessity occasions the taking down of an engine carefully watch the parts. If the manufacturer has not marked them for their respective places the motorist himself should do so. Take the case of the valves. Exhaust valves will not always interchange, and if one, at some time or other, has been ground in a little more than the one next to it, it is hardly likely to suit the cylinder. Apart from the fact that the seatings will not agree, there is naturally a slight difference in the length of the stem, which is enough, sometimes, to spoil the compression through the valve not seating properly.

If petrol drips from the carburettor when the car is standing with the engine stopped the needle valve connected with the float should be looked at. If pressing it down stops the dripping the float is too high. If the dripping persists the valve leaks and requires grinding. "Viator."

